# Gene Expression Profile of *Mycobacterium leprae* Contribution in the Pathology of Leprosy Neuropathy

**DOI:** 10.3389/fmed.2022.861586

**Published:** 2022-04-15

**Authors:** Beatriz Junqueira de Souza, Mayara Abud Mendes, Gilberto Marcelo Sperandio da Silva, Patrícia Sammarco-Rosa, Milton Ozorio de Moraes, Marcia Rodrigues Jardim, Euzenir Nunes Sarno, Roberto Olmo Pinheiro, Bruno Siqueira Mietto

**Affiliations:** ^1^Leprosy Laboratory, Oswaldo Cruz Institute, Fiocruz, Rio de Janeiro, Brazil; ^2^Clinical Research Laboratory in Chagas Disease, Evandro Chagas National Institute of Infectious Diseases, Fiocruz, Rio de Janeiro, Brazil; ^3^Laboratory Animal House, Lauro de Souza Lima Institute, Sao-Paulo, Brazil; ^4^Laboratory of Cell Biology, Institute of Biological Sciences, Federal University of Juiz de Fora, Juiz de Fora, Brazil

**Keywords:** leprosy, *Mycobacterium leprae*, peripheral nervous system, Schwann cell, host-pathogen interaction

## Abstract

Peripheral neuropathy is the main cause of physical disability in leprosy patients. Importantly, the extension and pattern of peripheral damage has been linked to how the host cell will respond against *Mycobacterium leprae* (*M. leprae*) infection, in particular, how the pathogen will establish infection in Schwann cells. Interestingly, viable and dead *M. leprae* have been linked to neuropathology of leprosy by distinct mechanisms. While viable *M. leprae* promotes transcriptional modifications that allow the bacteria to survive through the use of the host cell's internal machinery and the subvert of host metabolites, components of the dead bacteria are associated with the generation of a harmful nerve microenvironment. Therefore, understanding the pathognomonic characteristics mediated by viable and dead *M. leprae* are essential for elucidating leprosy disease and its associated reactional episodes. Moreover, the impact of the viable and dead bacteria in Schwann cells is largely unknown and their gene signature profiling has, as yet, been poorly explored. In this study, we analyzed the early differences in the expression profile of genes involved in peripheral neuropathy, dedifferentiation and plasticity, neural regeneration, and inflammation in human Schwann cells challenged with viable and dead *M. leprae*. We substantiated our findings by analyzing this genetic profiling in human nerve biopsies of leprosy and non-leprosy patients, with accompanied histopathological analysis. We observed that viable and dead bacteria distinctly modulate Schwann cell genes, with emphasis to viable bacilli upregulating transcripts related to glial cell plasticity, dedifferentiation and anti-inflammatory profile, while dead bacteria affected genes involved in neuropathy and pro-inflammatory response. In addition, dead bacteria also upregulated genes associated with nerve support, which expression profile was similar to those obtained from leprosy nerve biopsies. These findings suggest that early exposure to viable and dead bacteria may provoke Schwann cells to behave differentially, with far-reaching implications for the ongoing neuropathy seen in leprosy patients, where a mixture of active and non-active bacteria are found in the nerve microenvironment.

## Introduction

Leprosy neuropathy is a chronic neurological condition, caused by the infection of the nerve by its etiologic agents, *Mycobacterium leprae* and *Mycobacterium lepromatosis* (1–5). *M. leprae* infection provokes early pathological changes in the host cell that are, to some extent, associated with the late degenerative appearance of the infected nerves (6, 7). Schwann cells, the glial cells of the peripheral nervous system, are the preferable host for *M. leprae* entry, persistence, and replication within the nerve (8–11). Therefore, much attention has been given to the molecular and cellular alterations driven by leprosy bacilli once inside Schwann cells in order to identify the underlying reasons that culminate in the severe neuropathy seen in patients.

The immune response elicited in the nerve microenvironment against the bacilli is a key component that may lead to the distinct clinical manifestations (4). Infected Schwann cells produce a broad panel of inflammatory chemokines and cytokines, that accompanied with cell-mediated immune response, have been linked to the manifestation of neural pain and leprosy neuritis in patients (12–15). Of importance, this immunomodulation profile seen in infected Schwann cells was reported to occur before the reprogramming of the Schwann cells to the immature phenotype, highlighting the crucial role of the immune signaling network in the context of the early stages of *M. leprae* infection (16, 17). Additionally, *M. leprae* infection of Schwann cells has been associated with alterations in the glucose/lactate metabolic pathway (18, 19), lipid/cholesterol accumulation (20, 21), mitochondrial dysfunction (11), and myelin dismantling (22–24). Some of these changes were also confirmed in leprosy patients, and are suggested to cause the ongoing neuropathy and the observed tissue fibrosis and loss of nerve function experienced by leprosy individuals (14, 25). However, while these events may arise from modifications of the host Schwann cell's supportive function of the nerve, the accompanying changes in gene expression profile are largely unknown.

Studies have shown that the phenolic glycolipid 1 (PGL-1), a major *M. leprae* cell wall pathogenic component, is essential for *M. leprae* internalization into Schwann cells and has also been extensively attributed to induce pathology *in vitro* and in experimental infectious models (10, 23, 26). The understanding of the immunopathogenic mechanisms related to nerve damage in leprosy patients is pivotal for the development of new therapeutic strategies to control leprosy neuropathy. The treatment of nerve damage with steroids is effective but at least 40% of patients relapse and require a further course of steroids (27, 28).

Because leprosy neuropathy is an intricate complex disease, in which both viable and dead *M. leprae* may have a detrimental role for disease progression, it is necessary to fully understand and decipher the contribution of viable and dead bacteria in altering Schwann cell biology. For example, it was suggested that dead bacilli, unlike viable *M. leprae*, make Schwann cells susceptible to attack by killer cells (29). Moreover, dead bacteria and its components, such as lipoarabinomannan (LAM), were also reported to cause neural damage via modulation of the autophagic flux (30) and the complement attack of the nerve (31).

Despite these observations, the early effects of viable and dead *M. leprae* on the global Schwann cell gene expression profile that may be linked to primary neural leprosy are still largely unknown. Therefore, in the present study, we analyzed the expression profile of transcripts involved in neuropathy, glial cell plasticity, nerve repair, and the inflammatory network in leprosy and non-leprosy nerve biopsies and after challenging Schwann cells with viable and dead *M. leprae* independently. Our utmost goal was to provide novel evidence of how viable and dead bacteria modulate Schwann cell gene expression responses along with a detailed statistical correspondence to several histopathological findings commonly observed in nerve biopsies from leprosy and non-leprosy patients.

## Materials and Methods

### Human Nerve Biopsy

Nerve biopsy specimens from eight patients diagnosed with pure neural leprosy (PNL) were obtained from volunteers recruited at the Souza Araujo Outpatient Unit (Leprosy Laboratory, Oswaldo Cruz Institute, Oswaldo Cruz Foundation) (Table 1). Nerve biopsy fragments, as well their nerve sections, were available for histopathological staining and PCR analysis. For the present study, patients with PNL were selected who did not present any sign of nerve endoneurial fibrosis to ensure the chosen nerve specimens were in the early stages of leprosy neuropathy progression. This selection was made after analyzing the nerve section stained with the hematoxylin and eosin and Gomori trichrome stains under a light microscope following previously published protocol (6). Exclusion criteria were patients with coinfection, metabolic comorbidities such as diabetes, and signs of endoneurial fibrosis, pregnant women and patients under 18 years. For the control group, nerve biopsy specimens from three individuals who underwent brachial plexus surgery were kindly donated by the University Hospital Clementino Fraga Filho (HUCFF-UFRJ). This study was approved by the Oswaldo Cruz Foundation Ethics Committee (number of purports: 2.227.887).

**Table 1:** Clinical data from PNL patients included in the present study (*n* = 8).

**Age (years)**	**Gender**	**Leprosy clinical form**	**Leprosy reaction**	**Multidrug therapy**	**Physical disability level**
67	female	PNL	RR + Neuritis	No	0
26	male	PNL	RR + Neuritis	No	0
34	female	PNL	No	No	0
48	male	PNL	RR + Neuritis	No	0
47	female	PNL	RR	No	2
22	male	PNL	RR	No	2
22	female	PNL	No	No	0
48	female	PNL	No	No	0

*PNL, Pure Neural Leprosy; RR, Reverse Reaction*.

### Schwann Cell Culture

The human Schwann cell line, ST88-14, was used in the present study for the *in vitro* assays. Prior to the assays, cells were cultured in RPMI media (Gibco BRL, Grand Island, NY, USA) supplemented with 1% penicillin, 1% streptomycin, 2 mM L-glutamine, and 10% fetal bovine serum. The cells were maintained in a controlled environment at 37°C and 5% CO_2_. For the assays, ST88-14 cells were suspended in culture medium without penicillin-streptomycin and cultured at a density of 5 × 10^5^ cells/well on six-well culture plates. The cell culture was infected with viable *M. leprae*, gently donated by Lauro de Souza Lima Institute (Sao-Paulo, Brazil) or stimulated with dead (gamma-irradiated) *M. leprae*, obtained through BEI Resources (#NR-19326), at a multiplicity of infection (MOI) of 50 bacilli/cell (50:1). After 24 h of incubation, supernatants were harvested and kept frozen at −20°C until quantification of inflammatory chemokines and cytokines. Additionally, Schwann cell cultures were subjected to total RNA extraction procedures.

### RNA Extraction and RT-qPCR Array

Schwann cell cultures and nerve biopsy fragments were mechanically grinded and resuspended in 1 mL TRIzol (Gibco BRL) and RNA was obtained following the manufacturer's orientations and stored at −70°C until use. After, 10 ng of total RNA was reverse-transcribed to cDNAs using the Superscript III kit (Invitrogen, Carlsbad, CA, USA) and then amplified using the SYBR Green PCR Master Mix (Applied Biosystems, Foster City, CA, USA) or TaqMan assays (ThermoScientific). The GeneQuery™ Human Schwann cell PCR Primer library array kit (Realtime Primers, Elkins Park, PA, USA #GK096) was used to profile total gene expression in Schwann cells and human nerve biopsies. The full list of genes is available on https://www.sciencellonline.com/genequerytm-human-schwann-cell-biology-qpcr-array-kit.html” (accessed on 27th Jan 2022). The TaqMan Fast Universal PCR Master Mix and Human TaqMAn MGB-Probe assays (ThermoScientific), were used to determine mRNA expression of TNF (HS-99999043_m1), IL-23A (HS-0037334324_m1), CCL2 (HS-00234140_m1) and CXCL10 (HS-0017042_m1). The RT-qPCR array was performed in triplicate, and the amplifications were carried out in the ViiA7 Real-Time PCR System (Thermo Fisher Scientific Inc., Waltham, MA, USA). The ΔΔCT method (32) was used to analyze the obtained data after normalization using the endogenous control of the housekeeping gene *RPL13*, for SYBR Green analysis, or normalized using the housekeeping gene glyceraldehyde-3-phosphate dehydrogenase (GAPDH; HS-02758991_g1), for TaqMan assays.

### Enzyme-Linked Immunosorbent Assay

For cytokine/chemokine release evaluation, the supernatants from control ST88-14 cultures and *M. leprae* (viable or dead) infected ST88-14 cultures were harvested after 24 h and stored at −20°C until use. The following inflammatory mediators (TNF, TGF-β, IL-6, IL-8, IL-12, IL-10, MCP-1/CCL2, and IP-10/CXCL10) were quantified by ELISA technique following the manufacturer's orientations (R&D Systems, Minneapolis, MN, USA).

### Statistical Analysis

Analyses of the experiments were performed by unpaired *t*-test, Kruskal-Wallis test or one-way ANOVA. For all statistical analyses the value of *p* ≤ 0.05 was considered significant. Statistical analyses were performed using the GraphPad Prism version 8.0 software (GraphPad Software, San Diego, CA, USA). Alternatively, a correlogram graph was generated to evaluate the correlation between histopathological characteristics in nerve fragments and the pattern of gene expression. A Pearson's correlation was applied to each pair of variables present in the data. The correlation value varies between −1 and 1, with negative values implying the existence of negative correlation and positive values implying positive correlation. The strength of the correlation is measured by the proximity of the value to 1 or −1, with values closer to these suggesting stronger correlation. Each regression was performed using the gene expression as the response variable and initially both PCR and Acid Fast Bacilli (AFB) as covariates. Each coefficient had its statistical significance tested by the *t*-test for regression coefficients. In the cases where one of the variables was not statistically significant, the model was fitted again using only the statistically significant covariate. Finally, the model's goodness of fit was evaluated using R^2^, a statistical measure that evaluates how much of the variation on the response variable is explained by the covariates.

## Results

### Gene Profiling Analysis of Leprosy and Non-leprosy Nerve Biopsies

**Figure 1:**
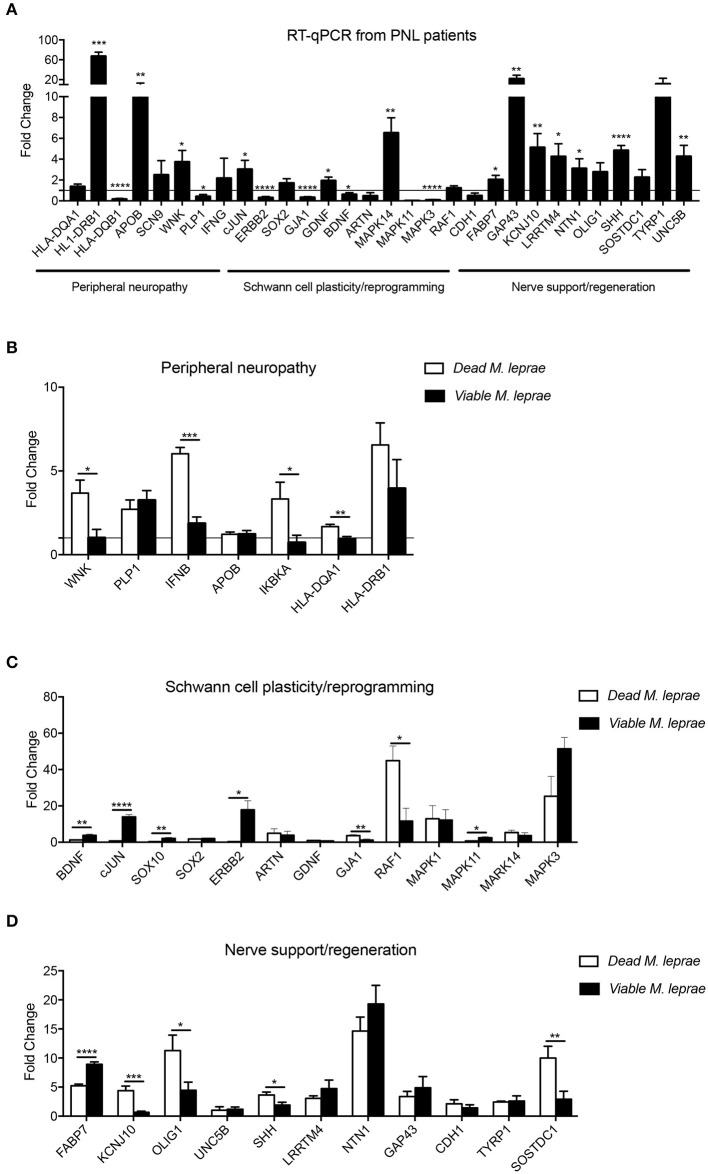
Expression pattern of genes involved in Schwann cell biology. Gene expression profile analysis of upregulated and downregulated mRNA transcripts from signaling pathways related to peripheral neuropathy, Schwann cell plasticity and regeneration support. Analysis were performed in human nerve biopsies from leprosy (**A**, black bars) and non-leprosy individuals (**A**, horizontal line) and in human Schwann cells **(B-D)** infected with dead *M. leprae* (white bars) and viable *M. leprae* (black bars); results are presented as mean ± SD from three to eight normalized independent biological replicates; *p < 0.05, **p < 0.01, ***p < 0.001, ****p < 0.0001.

In order to identify the molecular pathways related to primary neural leprosy (PNL), a Schwann cell biology PCR array was performed comparing gene expression in nerve fragments from PNL patients and non-leprosy controls. As illustrated in Figure 1A, differentially expressed patterns of genes related to peripheral neuropathy, Schwann cell plasticity/reprogramming, and nerve support could be observed. Regarding neuropathy-related genes, some targets were statistically elevated in nerves from PNL patients, such as *HLA-DRB1, APOB*, and *WNK*, while others were downregulated, including *HLA-DQB1* and *PLP1*. Interestingly, *HLA-DRB1*, previously reported to influence leprosy susceptibility (33), was upregulated 60-times more in leprosy nerves when compared to non-leprosy nerves. *APOB* and *WNK* were also augmented in leprosy nerves, by 10- and 4-times more, respectively.

By examining the mRNA profile involved in Schwann cell dedifferentiation, we observed that *cJUN, GDNF*, and *MAPK14* were statistically upregulated, while *ERBB2, GJA1, BDNF*, and *MAPK3* were significantly decreased in nerves from PNL patients. Although neurotrophins, such as BDNF, are required for axon regeneration, here we found that *BDNF* was downregulated in PNL patients, which is in agreement with a previous report that investigated neurotrophin expression in leprosy infection (34).

In summary, our PCR array analysis remarkably showed that most genes involved in nerve regeneration were significantly increased in leprosy nerve biopsies, possibly suggesting a continuous balance of nerve degeneration and an attempt to regrow during the course of infection *in vivo*.

### Viable and Dead *M. leprae* Distinctly Modulate Schwann Cell Functional Genes

**Figure 2:**
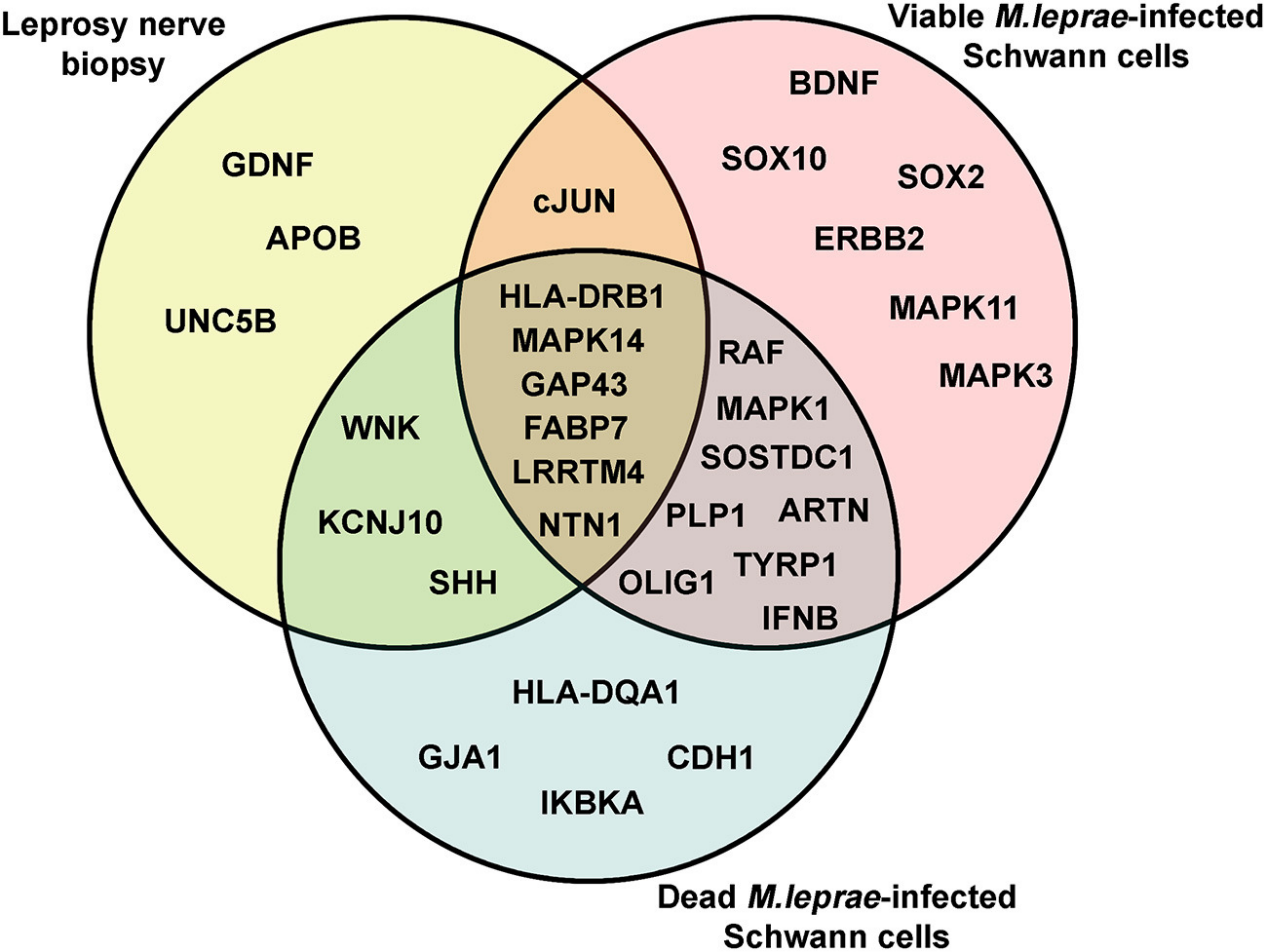
The Venn diagram of differentially upregulated genes related to Schwann cell biology genetic signature in leprosy. The Venn diagram was used to identify clusters of overlapping and non-overlapping mRNA transcripts, upregulated in leprosy nerve biopsies and in Schwann cells infected with viable and dead *M. leprae*.

Neural damage in leprosy has been associated with the role played by viable and dead bacteria after being in contact with Schwann cells (10, 35). Therefore, we were interested in exploring the early effect of *M. leprae* infection on Schwann cell biology genes. For that, we infected human Schwann cells with viable and dead *M. leprae* and screened changes in the mRNA levels after 24 h of infection using the Schwann cell biology PCR array. As observed in Figures 1B-D, dead *M. leprae* increased the expression of *WNK, IFNB, IKBKA*, and *HLA-DQA1* (genes related to neuropathy), in addition to *GJA1* and *RAF1* (for Schwann cell reprogramming) and *KCNJ10, OLIG1, SHH*, and *SOSTDC1* (for neural regeneration). Interestingly, viable *M. leprae* appeared to modulate genes related to Schwann cell plasticity and dedifferentiation, such as *BDNF, cJUN, SOX10, ERBB2*, and *MAPK11*. In summary, this first set of analysis points to the notion that dead *M. leprae* induces greater expression of peripheral neuropathy and nerve regeneration support genes whereas viable *M. leprae* acts by modulating genes related to Schwann cell plasticity and dedifferentiation. We have summarized this gene intersection in a Venn diagram (Figure 2).

### Inflammatory Network Analysis Suggests That Dead and Viable *M. leprae* Increase CCL2 Expression

**Figure 3:**
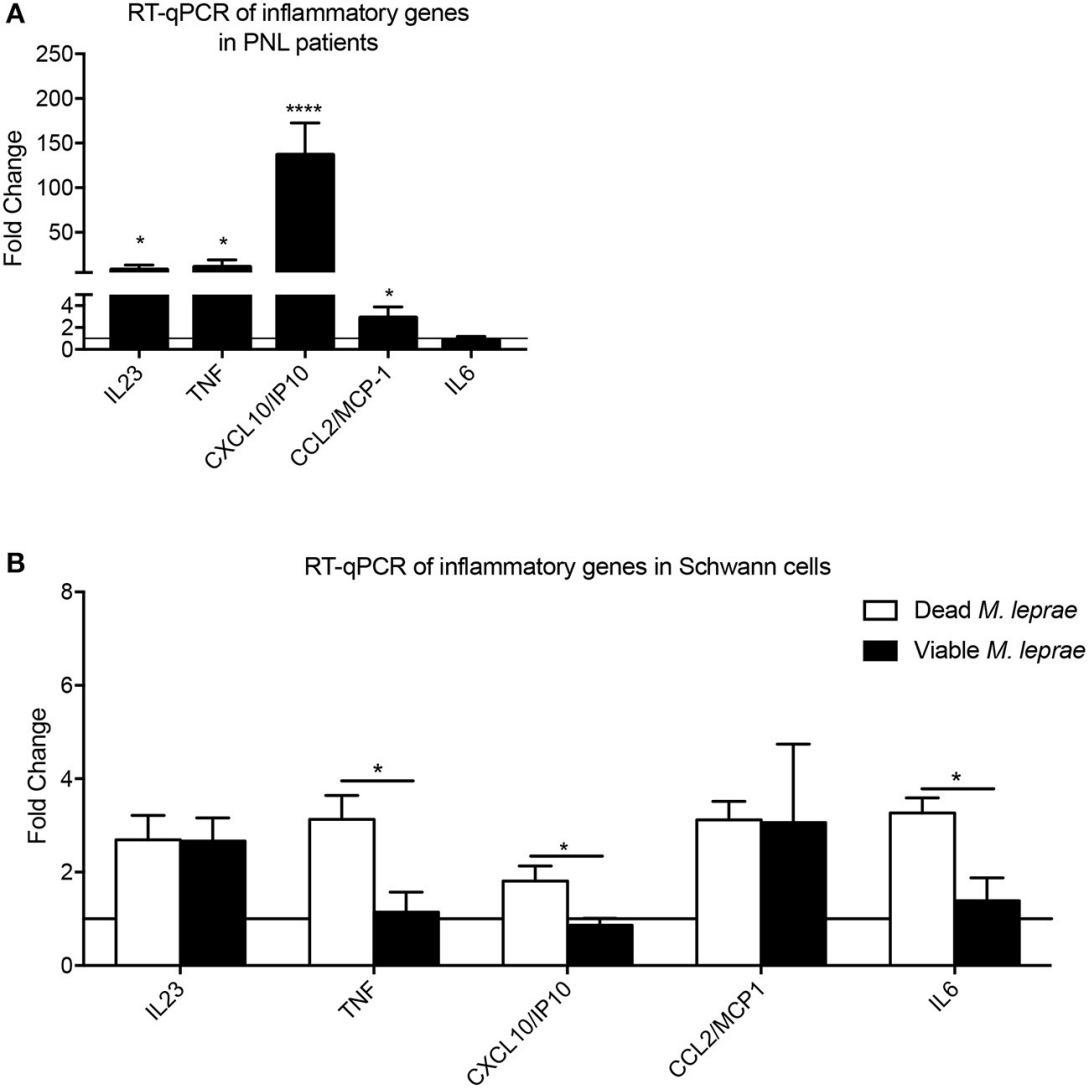
Analysis of inflammatory network in leprosy infection. Fold-change measurement of inflammatory cytokines/chemokines mRNA transcripts in human nerve biopsies from leprosy (**A**, black bars) and non-leprosy patients (**A**, horizontal line) and in human Schwann cells **(B)** infected with dead *M. leprae* (white bars) and viable *M. leprae* (black bars); results are presented as mean ± SD from three to eight normalized independent biological replicates; *p < 0.05, ****p < 0.0001.

We next aimed to analyze the inflammatory network profile in leprosy and non-leprosy nerve biopsies, as well as *in vitro*, using Schwann cells challenged with viable and dead bacteria. The human nerve analysis indicated that *IL23, TNF, CXCL10*, and *CCL2* were increased in PNL patients when compared with control biopsies (Figure 3A). In addition, when we analyzed changes in Schwann cells *in vitro*, we observed that dead *M. leprae* induced a higher expression of *TNF, CXCL10*, and *IL6* in Schwann cells when compared with those infected with viable bacilli (Figure 3B). Furthermore, cells infected with viable *M. leprae* increased *IL23* and *CCL2* expression when compared to non-stimulated cultures. These results support the notion that dead bacteria are likely involved in the induction of a pro-inflammatory profile, suggesting that such pro-inflammatory mediators, in the context of neural involvement shown by the biopsies, are induced by dead bacteria present at the site of infection.

### Viable and Dead *M. leprae* Promote a Distinct Inflammatory Response in Schwann Cells

**Figure 4:**
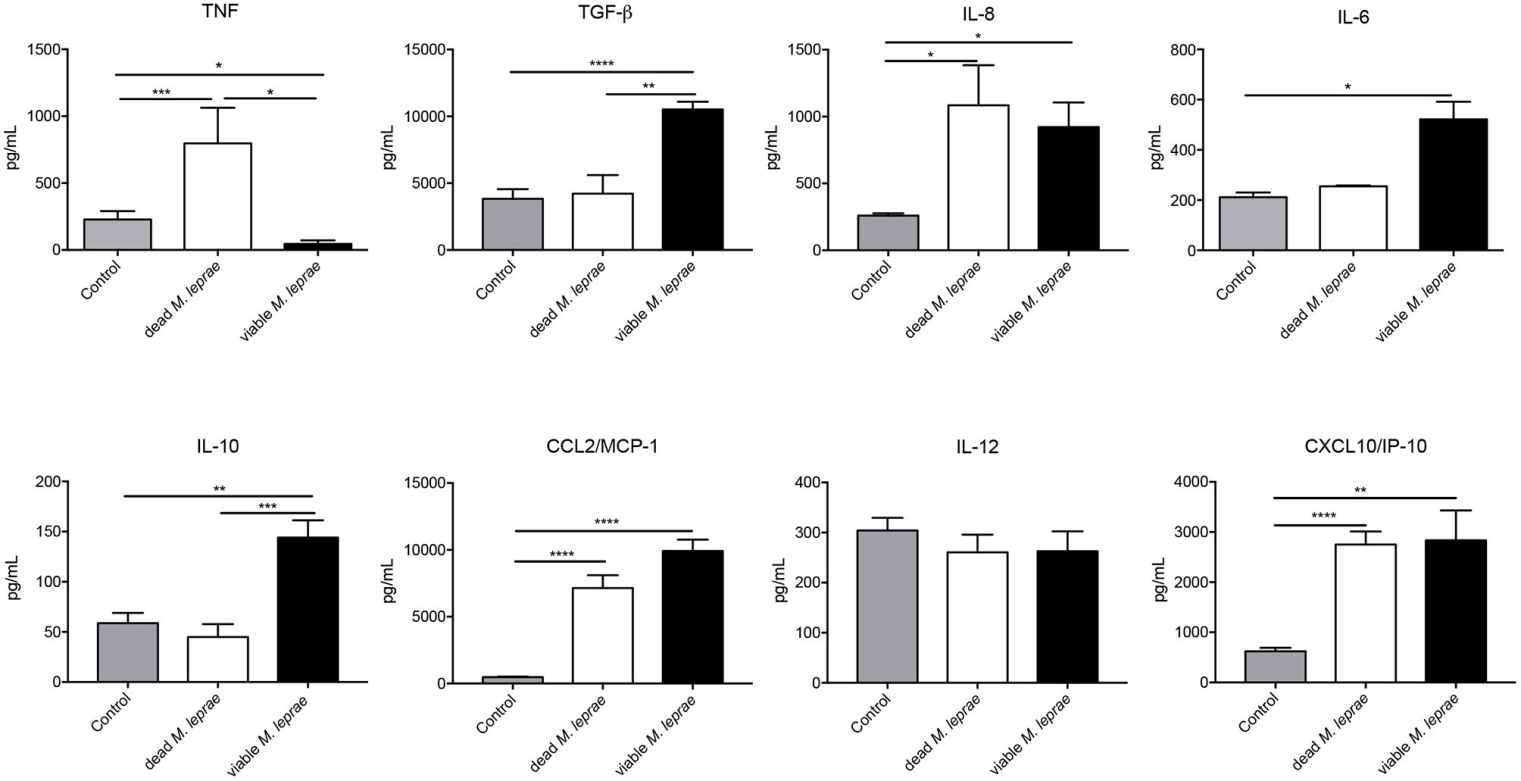
Differentially pattern of cytokine/chemokine release after viable and dead *M. leprae* infection in human Schwann cells. Two-side sandwich ELISA showing the pattern secretion of pro- and anti-inflammatory cytokines and chemokines, in human Schwann cells control (gray bars) and after infection with dead *M. leprae* (white bars) and viable *M. leprae* (black bars), at 24 h after stimuli *in vitro*, results are shown as mean ± SD from three to eight normalized independent biological replicates; *p < 0.05, **p < 0.01, ***p < 0.001, ****p < 0.0001.

We substantiated our transcriptional analysis by evaluating the cytokine network production in Schwann cells challenged with viable and dead bacilli. For that, we performed analysis of cytokine levels in 24-h supernatants, and found that dead *M. leprae* caused an increase in the TNF, IL-8, MCP-1/CCL-2, and CXCL-10 levels in comparison to non-stimulated control cultures (Figure 4). Conversely, viable *M. leprae* infection led to increased TGF-β, IL-8, IL-6, IL-10, MCP-1/CCL2, and CXCL-10 in comparison to non-stimulated controls. We also observed a statistical reduction in TNF levels after viable M. leprae infection when compared to dead stimulated cultures and controls. Together, these results suggest that dead *M. leprae* induces pro-inflammatory mediators in human Schwann cells, whereas viable *M. leprae* preferably promote anti-inflammatory cytokines like IL-10 and TGF-β and reduced TNF production (Figure 4).

### *CCL2* Correlates With Decreased Fiber in Histopathology, AFB and PCR From Nerve Fragments

**Figure 5:**
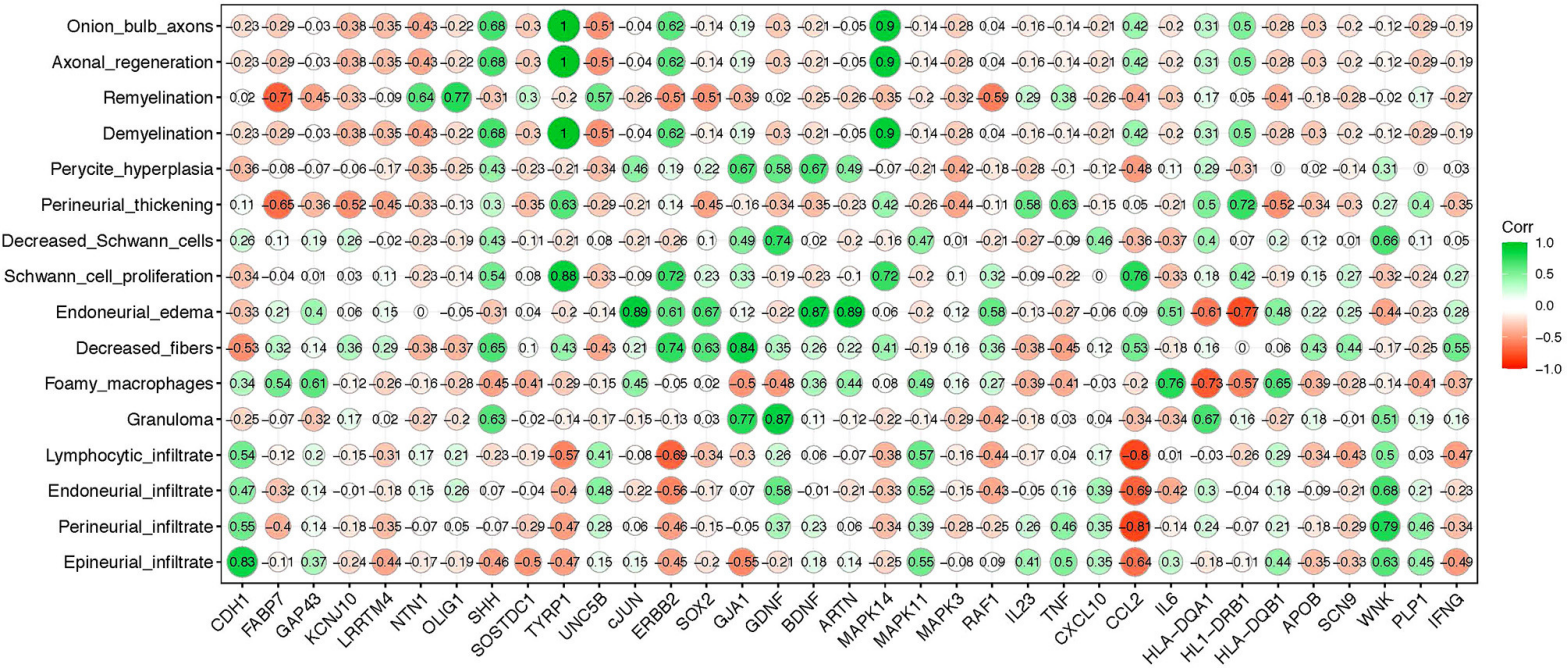
Correlation matrix between leprosy histopathological alterations and gene expression profile in PNL patients. The y-axis indicates major nerve histopathological alterations observed in leprosy patients; the x-axis shows Schwann cell genes evaluated in PNL patients. The green color (closer to 1 value) means stronger correlation while the red color (closer to −1 value) means weaker correlation between the gene expression and the specific histopathological alteration, respectively.

We next applied a correlation matrix to analyze a potential relationship of histopathological findings and changes in gene profiling of Schwann cells in order to evaluate top molecular signature candidates during the early stages of nerve damage in leprosy patients. The overall nerve pathological findings are listed on the y-axis of Figure 5. Using this correlogram, we observed that, onion bulb axons, axonal regeneration, demyelination, and Schwann cell proliferation were positively correlated with *SHH, TYRP1, ERBB2*, and *MAPK14* genes. With regards to remyelination appearance, this was positively correlated with *NTN1, OLIG1*, and *UNC5B* genes. Foamy macrophages correlated with *IL6* and granulomas were positively correlated with *GDNF, GJA1*, and *SHH* (Figure 5). *CCL2* was negatively correlated with lymphocytic, perineurial, and epineurial infiltrate and positively correlated with decreased fibers and Schwann cell proliferation (Figure 5).

Acid Fast Bacilli (AFB) staining in nerve lesions and positive PCR are two commonly used tools for diagnosing PNL at clinics. Thus, we next searched for a potential association between the presence of the bacilli in nerve and changes in gene expression. It was statistically relevant that the expression of *CCL2* and *CDH1* positively correlated with AFB, while, CCL2 and GDNF expression negatively correlated with PCR (Table 2).

**Table 2:** Linear regression between *M. leprae* PCR and AFB values against the global gene expression.

**Variable**	**Estimative**	**Standard deviation**	***P*-value**
**CCL2**
Intercept	0.622	1.112	0.5998
FAB	5.247	1.435	0.0147^*^
PCR	−4.445	1.284	0.0180^*^
R^2^	0.778
**CDH1**
Intercept	0.108	0.253	0.6818
BFAB	1.014	0.412	0.0493^*^
R^2^	0.502
**GDNF**
Intercept	3.119	0.503	0.000815^***^
PCR	−1.596	0.581	0.033469^*^
R^2^	0.557

*Among all the genes evaluated, CCL2, CDH1, and GDNF presented a statistical significance with AFB and/or PCR*.

* *p < 0.05*,

*** *p < 0.001*.

## Discussion

The comprehension of Schwann cell-*M. leprae* pathological interactions is vital to fully understand disease progression in experimental models as well as in leprosy patients. Moreover, how viable and dead bacteria affect Schwann cell biology is largely unknown and poorly explored in the field. Therefore, in the present work, we used the *in vitro* host-pathogen interaction model to determine how viable and dead bacteria modify the global gene expression profile as soon as the bacteria comes in contact with the Schwann cells. Furthermore, we extended these early changes in the Schwann cell gene profile in response to infection to the analysis of patient nerve biopsies, establishing a potential link to the early response pattern during the disease progression in leprosy individuals.

We initially performed an exploratory, broad gene expression analysis of Schwann cells challenged with viable or dead bacteria, along with data obtained from nerve biopsies. The following families of genes involved in the nerve response to injury were investigated: (i) peripheral neuropathy, (ii) inflammatory network, (iii) Schwann cell plasticity/dedifferentiation, and (iv) nerve regeneration support.

Among the global gene expression, *HLA-DRB1, MAPK14, GAP43, FABP7, NTN1*, and *LRRTM4* were upregulated in nerve biopsies from PNL patients and in viable and dead *M. leprae*-infected Schwann cells, in comparison with the respective control groups (Figure 2). It has been described that human leukocyte antigen (*HLA*) alleles affect the host immune response against *M. leprae* (36, 37). *HLA-DRB1* is one of the most upregulated genes in multiple sclerosis, having a special role in inducing demyelination (38). Moreover, this transcript is consistently related to the tuberculoid leprosy (T-Lep) clinical form, while *HLA-DQB1* has a strong relation with the L-Lep clinical form (39). In the present study, *HLA-DQB1* was downregulated in PNL patients, in comparison with the uninfected nerve specimens (Figure 1A). Additionally, receptors for the axon guidance molecule, netrin-1 (*NTN1*), are expressed by Schwann cells and play a role in peripheral regeneration and induce the regeneration phenotype (40).

When evaluating the set of genes related to Schwann cell plasticity and dedifferentiation, some serine/threonine kinases, such as mitogen-activated protein kinase 14 (*MAPK14*), widely known for its role at the inflammasome pathways in the neuroglia (41), were found to be upregulated in PNL patients. However, there is a lack of information regarding *MAPK14* in leprosy. *GAP43*, the major protein of the axon growth-cone that plays a role in axon growth (42) and regeneration was also upregulated, which suggests a tentative role for axonal regrowth in the injured infected nerve. *SHH, WNK*, and *KCNJ10* were upregulated in leprosy nerve biopsies and in dead *M. leprae*-infected Schwann cells (Figure 2). The sonic hedgehog gene (*SHH*), which has been previously reported in nerve damage (43, 44), may also trigger regeneration and induce Schwann cell proliferation, as an attempt to counteract the harm caused by the bacilli (44). *KCNJ10* encodes the inward-rectifying potassium channel (Kir4.1). Alteration of *KCNJ10* gene expression is related to neuropathies, such as Huntington's disease East/SeSAME syndrome, by elevating the extracellular K^(+)^, which consequently leads to abnormal neuron excitability (45, 46). It was demonstrated in mice that inflammation can silence (Kir4.1) channels, leading to hyperalgesia and trigeminal pain (47). Despite the absence of studies relating the *KCNJ10* gene and *M. leprae* infection, it seems this may be a potential pathway to be explored for leprosy neuritis in the future. *cJUN* was the only gene statistically upregulated in PNL biopsies and viable *M. leprae*-infected Schwann cells in comparison to the respective controls (Figure 2). *cJUN* is a master Schwann cell regulator involved in the transition of a differentiated phenotype toward a progenitor/stem-cell like stage (48) and has also previously been reported to be upregulated, among other developmental and neural crest genes, during *M. leprae* infection in mouse Schwann cells (35).

*PLP1, SOSTDC1, RAF1, ARTN, OLIG1, MAPK1, IFNB*, and *TYRP1* were upregulated in viable and dead *M. leprae*-infected Schwann cells, but not in the PNL biopsies. The tyrosinase-related protein 1 (*TYRP1*) plays a role in the melanin biosynthetic pathway, being mostly expressed by melanocytes (49). It has been documented that leprosy patients may present hyper or de-pigmented lesions due to a defective transfer of melanin (50). The melanocytes have been recently associated with the innate immune response, by producing inflammatory cytokines, such as IL-1β (50). But the role of the *TYRP1* pathway in the peripheral nerve requires further investigation. It was documented that interferon beta (*IFNB*) is increased during cell stress conditions, but the lack of this protein leads to neuroinflammation (51, 52). *IFNB* is essential to start the cell fate pathway driven by *NUPR1*, a gene signature that contributes to a progressive infection in human cells (53). Oligodendrocyte transcription factor 1 (*OLIG1*) is an important protein of the remyelination phenotype, usually upregulated after a disturbance in the cell microenvironment (54). It is well described that during diabetic neuropathy, the lack of insulin leads to the demyelination phenotype due to a downregulation of *OLIG1* (54). On the other hand, in this study, some genes like *MAPK3* (*ERK1*), an important extracellular signal-regulated kinase and Schwann cell migration and proliferation molecule (55) was downregulated in PNL patients. Likewise, ErbB2, a well-known demyelination inducer and Connexin43—*GJA1—*were also significantly reduced in PNL patients. Despite the lack of information about *GJA1* in leprosy, this has been described in neural impairment, like in the pathogenesis of Alzheimer's disease, where GJA1 downregulation leads to reduced levels of ApoE (56, 57).

While we know that the polarity of leprosy can result from the host's inflammatory response profile, multiple features of neurological involvement may also occur. For example, polymorphisms in certain genes such as IL10, ninjurin 1 and TNF have been associated with leprosy susceptibility (58–60).

In the present work, we were able to correlate the pattern of histopathological impairments with alteration in the gene expression profile induced by *M. leprae* infection. The correlogram analysis demonstrated, for the first time, a statistically significant correlation between the gene signature from the early stages—before fibrosis—and the histopathological alterations of the nerve damage in leprosy neuropathy. Regarding the histopathological features, *TYRP1, SHH*, and *MAPK14* expression were strongly correlated to onion bulb axons, axon regeneration, demyelination, and Schwann cell proliferation, while *MCP1/CCL2* was strongly negatively correlated with nerve inflammatory infiltrates: epineurial, endoneurial, perineurial, and lymphocytic infiltrates. It is important to realize that the weak correlation with endoneurial, perineurial, and epineurial fibrosis was due to the inclusion criteria for this study, which selected patients with no signs of fibrosis.

The biserial correlation demonstrated that the monocyte chemoattractant protein 1 (*MCP1/CCL2*) was positively correlated with AFB and negatively correlated with PCR, which strongly indicates the correlation of increased expression of this gene by viable bacteria. This finding corroborates a previous study by our group, which showed activation of the ESX-1 mycobacterial system by the viable *M. leprae*, leading to the activation of the *OASL* gene and the induction of *CCL2*, impairing the host bactericidal response, which was not observed with dead *M. leprae* stimuli in THP-1 cells (61). More than that, independently, Schwann cells are responsible for the triggering response of nerve damage through initiating the clearance of the debris by myelinophagy, followed by macrophage recruitment, which is especially regulated by CCL2 (62). In this sense, we see the relevance of *CCL2* gene activation not only as a protective mechanism in the maintenance of mycobacterial viability, but also as a biological marker indicative of positive AFB. CCL2 has been described as one of the innate immunity genes immediately activated in the context of infection *in vitro* (35). Thus, despite the relevant findings, there is still a way to go in terms of understanding the role of this chemokine in infection and nerve damage during leprosy.

To strengthen the data obtained from the gene signature of *M. leprae* infection, we evaluated the immunomodulatory profile secreted by Schwann cells without infection and challenged with viable and dead *M. leprae*. We observed a pro-inflammatory profile elicited by the dead bacilli, while an anti-inflammatory microenvironment appeared to be promoted by infection with the viable bacteria. Studies have shown that macrophage infection by viable *M. leprae* leads to a regulatory T cell response rather than a cytotoxic T cell response, which contributes to the persistence of the infection in the host (63, 64). This characteristic is already well demonstrated in patients with L-lep, which leads to a high bacillary load. In the present work, we demonstrated that Schwann cells also behave in a way to induce the anti- or pro-inflammatory phenotype according to the bacilli viability. Since in reactional episodes there is a mixture of viable and dead bacilli, it is interesting to point out the role of the Schwann cells, and not only the macrophages, as modulators of the reaction process in the nerve, which can even lead to leprosy neuritis (14).

## Conclusion

In the present study, we identified the early impact of viable and dead bacteria, independently, on modifying the gene expression profile of human Schwann cells *in vitro*. We also described a molecular signature associated with neural damage in early stages of pure neural leprosy from patients (i.e., *HLA-DRB1, MAPK14, GAP43, FABP7, NTN1*, and *LRRTM4*). Leprosy is a complex intricate disease and the identification of this genetic profiling may contribute for the fully understanding of leprosy neuropathy pathogenesis with the long-term goal of identifying these pathways as targets for the development of effective therapeutic strategies. We acknowledge the relatively limited number of human nerve samples, as in our experimental design we narrowed our analysis to nerve biopsies of leprosy patients with no signs of neural fibrosis and at the initial stages of neural damage. Therefore, future work with additional nerve samples from leprosy individuals, at distinct clinical stages, are important to advance this host-pathogen interactions and associated genetic analysis in larger cohorts. In summary, these results open new perspectives for the understanding of the genetic signature of neural commitment in leprosy disease.

## Data Availability Statement

The data that support the findings of this study are available from the corresponding author upon reasonable request.

## Ethics Statement

The studies involving human participants were reviewed and approved by Oswaldo Cruz Foundation Ethics Committee (number of purports: 2.227.887). The Ethics Committee waived the requirement of written informed consent for participation.

## Author Contributions

BS, RP, and BM designed and carried out experiments, collected and interpreted data, and prepared illustrations. BS, GS, RP, and BM analyzed the data. BS, MM, GS, PS-R, MOM, MJ, ES, RP, and BM reviewed and edited the manuscript. ES, RP, and BM conceptualized and designed experiments, supervised the study, interpreted data, and obtained funding. BS, RP, and BM wrote the manuscript. PS-R provided viable *M. leprae*. All authors read and approved the final version of the manuscript.

## Funding

This study was supported by the Coordenação de Aperfeiçoamento de Pessoal de Nível Superior (CAPES), Fundação Carlos Chagas Filho de Amparo à Pesquisa do Estado do Rio de Janeiro (FAPERJ), Fundação Oswaldo Cruz (Fiocruz) and Conselho Nacional de Desenvolvimento Científico e Tecnológico (CNPq). Dead *M. leprae* was obtained through BEI Resources, NIAID, NIH: *Mycobacterium leprae*, Strain NHDP, Gamma-Irradiated Whole Cells (lyophilized), NR-19326.

## Conflict of Interest

The authors declare that the research was conducted in the absence of any commercial or financial relationships that could be construed as a potential conflict of interest.

## Publisher's Note

All claims expressed in this article are solely those of the authors and do not necessarily represent those of their affiliated organizations, or those of the publisher, the editors and the reviewers. Any product that may be evaluated in this article, or claim that may be made by its manufacturer, is not guaranteed or endorsed by the publisher.
